# Proteomics and bioinformatics analyses identify novel cellular roles outside mitochondrial function for human miro GTPases

**DOI:** 10.1007/s11010-018-3389-6

**Published:** 2018-06-25

**Authors:** Laura J. Kay, Vartul Sangal, Gary W. Black, Meera Soundararajan

**Affiliations:** Department of Applied Sciences, Faculty of Health and Life Sciences, Northumbria University, Newcastle, NE1 8ST UK

**Keywords:** Miro1, Miro2, Atypical GTPase, Mitochondrial function, Proteomics, Neurodegeneration, RhoC, Type III secretion system, VopE, Endoplasmic reticulum signalling

## Abstract

The human Miro GTPases (hMiros) have recently emerged as important mediators of mitochondrial transport and may significantly contribute to the development of disorders such as Alzheimer’s and schizophrenia. The hMiros represent two highly atypical members of the Ras superfamily, and exhibit several unique features: the presence of a GTPase domain at both the N-terminus and C-terminus, the presence of two calcium-binding EF-hand domains and localisation to the mitochondrial outer membrane. Here, elucidation of Miro GTPase signalling pathway components was achieved through the use of molecular biology, cell culture techniques and proteomics. An investigation of this kind has not been performed previously; we hoped, through these techniques, to enable the profiling and identification of pathways regulated by the human Miro GTPases. The results indicate several novel putative interaction partners for hMiro1 and hMiro2, including numerous proteins previously implicated in neurodegenerative pathways and the development of schizophrenia. Furthermore, we show that the N-terminal GTPase domain appears to fine-tune hMiro signalling, with GTP-bound versions of this domain associated with a diverse range of interaction partners in comparison to corresponding GDP-bound versions. Recent evidences suggest that human Miros participate in host–pathogen interactions with *Vibrio Cholerae* type III secretion proteins. We have undertaken a bioinformatics investigation to identify novel pathogenic effectors that might interact with Miros.

**Electronic supplementary material:**

The online version of this article (10.1007/s11010-018-3389-6) contains supplementary material, which is available to authorized users.

## Introduction

The Ras superfamily of small guanosine triphosphatases (GTPases), named after one of the most extensively studied oncogenes in human carcinogenesis, Ras, comprises over 150 distinct human members across six sub-families: Ras, Rho, Rab, Ran, Arf and Miro. Typically, these so-called ‘small’ GTPases are monomeric in nature, containing a canonical ~ 21 kDa catalytic ‘G domain’ [1, 2]. This G domain enables the hydrolytic degradation of GTP to GDP and is central to the function of Ras superfamily enzymes, enabling them to act as binary molecular switches within a diverse range of signalling pathways [3, 4]. Typically, these GTPases are ‘on’ (able to activate downstream signalling pathways) when GTP bound, and correspondingly ‘off’ (unable to activate downstream signalling pathways) when GDP bound, with this switch between ‘on’ and ‘off’ states tightly regulated by distinct groups of modulatory enzymes.

**Fig. 1:**
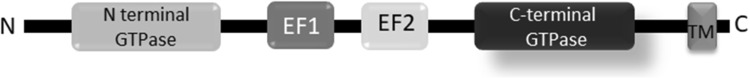
Schematic diagram of hMiro1 and hMiro2 GTPase domain architecture. Amino and carboxy terminus of the open reading frame shown using N and C. The transmembrane region responsible for localising to the mitochondrial outer membrane is depicted as TM

Initially characterised as atypical Rho GTPases [1], the Miro GTPases (‘mitochondrial Rho GTPases’) are so unusual that it is now considered appropriate to classify them into a separate sub-family: the Miro sub-family [2–4]. The Miro GTPases are evolutionarily conserved and first evolutionally appear in yeast [5, 6], with two genes encoding Miro GTPases present in humans: hMiro1 and hMiro2 (also referred to as RhoT1 and RhoT2). Both human Miros consist of 618 amino acid residues, sharing 60% amino acid identity [1]. As schematically illustrated in Fig. 1, the Miro GTPases are composed of two GTPase domains flanked by a pair of Ca^2+^-binding EF hand domains [1, 3, 5, 7–9]. Anchored to the mitochondrial outer membrane (MOM) by means of a C-terminal transmembrane sequence [10], the Miro proteins are accessible to the cytoplasm, where they act in an adaptor complex for mitochondrial movement and appear to be involved in a variety of mitochondrial-related processes including mitochondrial transportation, motility, morphology and homeostasis [7, 11–14].

Pioneering work in mammalian cell lines functionally implicated the hMiros in apoptotic pathways and mitochondrial dynamics. Later, abrogation of the single Miro homologue in Drosophila (dMiro) produced flies with small larvae size exhibiting symptoms associated with motor neurone disease: progressive locomotor defects culminating in early death. The effects of dMiro ablation were corrected upon reconstitution of neuronal dMiro, albeit not muscular dMiro, indicating a distinct importance for the Miro GTPases in neuronal health and pathogenesis. This link was reinforced by subsequent studies in mice and mammalian neuronal cell culture, showing that mitochondrial movement was severely impaired in neuronal cells upon Miro knockdown, and that Miro appeared necessary for correct prenatal innervation of the lungs during development in mice. Furthermore, several interaction partners for the mammalian Miros have recently been identified that implicate these remarkable enzymes in several serious pathologies including cancer, Parkinson’s disease (PD), Alzheimer’s disease (AD), ALS and schizophrenia.

Recent scientific investigations have clearly identified Miros’ influence on cell migration and proliferation through suppression of SMAD4 in pancreatic cells [15]. When chromosome partitioning and organelle segregation were studied, it was clearly shown that mitotic distribution of the mitochondrial network was mediated by Miro along with Cenp-F. Further Vibrio cholerae Type 3 secretion system effector VopE that localises to mitochondria during infection has been shown to act as a specific GTPase-activating protein to interfere with the function of mitochondrial Rho GTPases Miro1 and Miro2 [16].

Although Miro interaction partners continue to emerge, to our knowledge no high-throughput mass spectrometry-based proteomic analyses have been performed to evaluate the effects of the hMiro GTPase domain states on intracellular signalling pathway activities. The effect of the hMiro1 and hMiro2 N-terminal GTPase domain state produces distinct phenotypes visible through microscopy, though proteomic changes associated with these phenotypes have not been explored. A more complete understanding of the proteins expressed under constitutively active or dominant negative Miro N-terminal GTPase domain would serve as a valuable resource for understanding if, and how, this GTPase domain may influence or fine-tune intracellular signalling pathways. Towards this aim, we used a high-resolution accurate—mass Q-Exactive™ Hybrid Quadrupole-Orbitrap Mass Spectrometer to perform proteomic analyses of mammalian COS7 cells manipulated to overexpress either hMiro1 or hMiro2 N-terminal GTPase mutants. We identified hundreds of proteins for the first time unique to each test condition. When cross-referenced against bioinformatical resources, 13 unique proteins of interest were identified for hMiro1 signalling and 24 unique proteins of interest were identified for hMiro2 signalling. The results provide a proof-of-concept for studying Miro GTPase signalling pathways in this manner, suggesting that the state of the N-terminal GTPase domain is able to fine tune Miro signalling in mammalian cells.

## Results and discussion

**Fig. 2:**
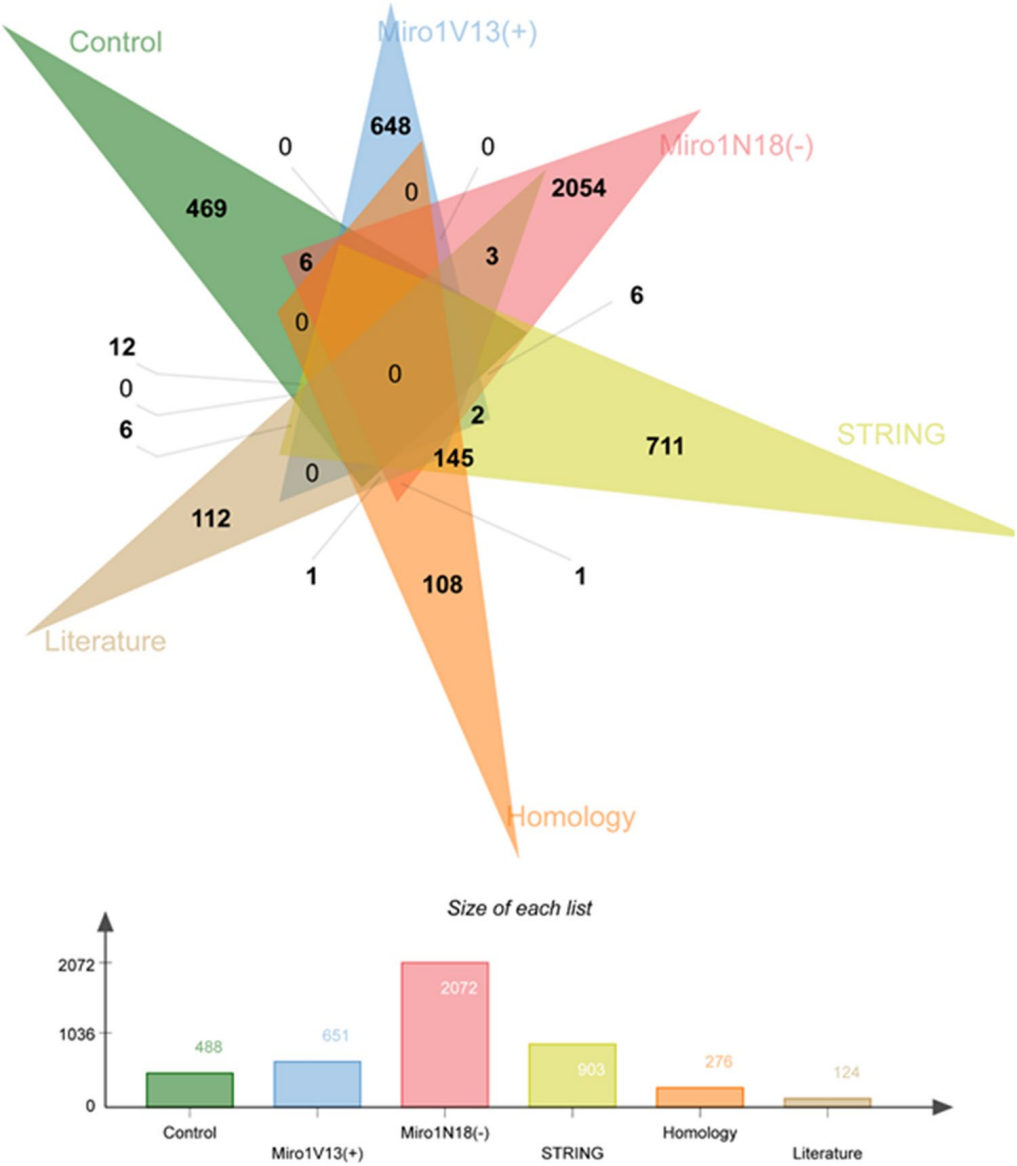
Number of proteins identified for each hMiro1 condition, cross-referenced against bioinformatics sources. The differentially expressed proteins identified for Miro1 constitutively active V13 and dominant negative N18 mutants against the control is given which is cross referenced with STRING bioinformatics tool. This is further cross referenced with literature reported and Miro homologues in lower organisms. The total differentially expressed proteins identified by fold change are given in each of the arm and the overlapping number of proteins between each species is also given

**Fig. 3:**
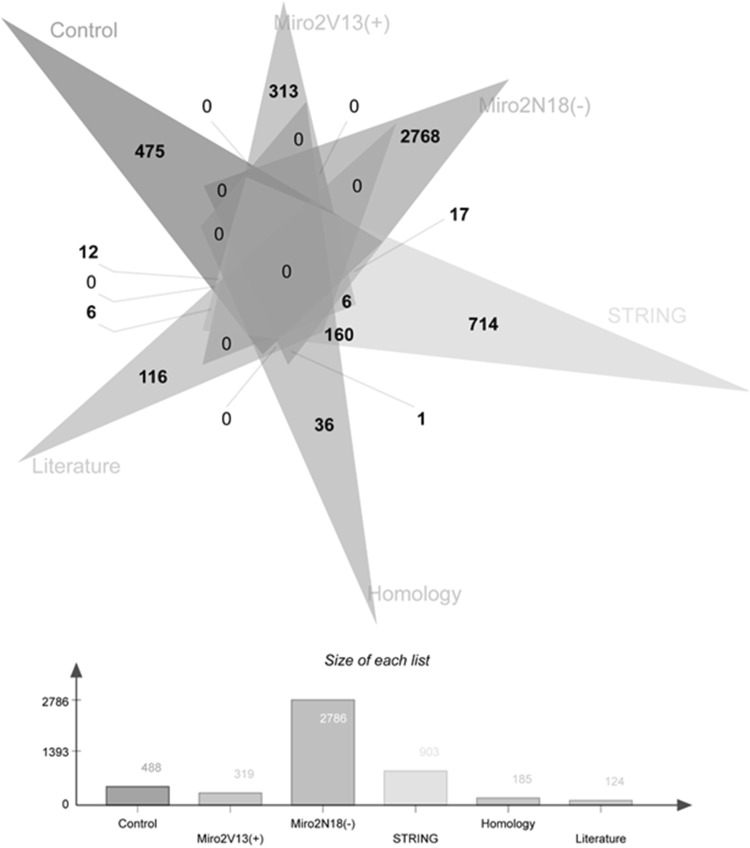
Number of proteins identified for each hMiro2 condition, cross-referenced against bioinformatics sources. The differentially expressed proteins identified for Miro2 constitutively active V13 mutant and dominant negative N18 mutants against the control are given which are cross referenced with STRING bioinformatics tool. This is further cross referenced with literature reported and Miro homologues in lower organisms. The total differentially expressed proteins identified by fold change is given in each of the arm and the overlapping number of proteins between each species is also given

LC-MS/MS analysis of control, V13 (constitutively active at the N-terminal GTPase domain) and N18 (dominant negative at the N-terminal GTPase domain) hMiro1 and hMiro2 samples resulted in the identification of the following numbers of unique proteins associated with each condition: 693 (control), 506 (hMiro1 V13), 429 (hMiro1 N18), 644 (hMiro2 V13) and 855 (hMiro2 N18). Of these proteins, more than 80% were identified with multiple peptides successfully matched. Complete lists of the peptides and proteins are provided in the ESI Table S1. We compared the proteomic data with protein–protein interaction predictions obtained from the STRING 2.0 protein–protein interaction prediction database, which integrates several different prediction algorithms, in addition to automated literature mining and surveying curated databases of known interactors, to generate networks of known and predicted protein interactors for defined protein entries. Additionally, we compared our proteomic data to known Miro interactors defined by manual literature searches, and by STRING 2.0 outputs for the three closest homologues of hMiro1 and hMiro2. The outputs for STRING 2.0 and the manual literature data are provided in the ESI. When results were cross-referenced against all bioinformatic sources, 13 unique proteins associated with hMiro1 mutants were identified (3 for V13 and 10 for N18 mutants) and 24 unique proteins associated with hMiro2 mutants were identified (for 6 V13 and 18 for N18 mutants), as shown in Figs. 2 and 3, respectively. Lists of these unique proteins are provided as Table 1 (hMiro1) and Table 2 (hMiro2). Analysis of the differentially expressed proteins clearly shows involvement of Miros in cellular processes such as mitochondrial movement, neuronal enriched proteins, ER related and developmentally implicated proteins that are reported in literature in varying extent. Conversely, very novel cellular processes such as protein expression, endocytosis, various metabolic processes, nucleotide biosynthesis apoptosis and Anaemia-related class of proteins are absolutely novel. A more detailed chart, displaying the function of proteins associated with either the V13 (constitutively active) or N18 (dominant negative) versions of hMiro1 and hMiro2, is shown in Fig. 5.

**Table 1:** Identification of 13 proteins of interest related to hMiro1 signalling

Identifier	Protein function	STRING score
Miro1V13(+)/STRING		
GPAT, ATASE, PRAT	Regulatory allosteric enzyme; catalyses the first step of *de novo* purine nucleotide biosynthetic pathway	0.999
SUMO3, HSMT3, smt3A	Member of the small ubiquitin-related modifier (SUMO) family; becomes covalently conjugated to other proteins via post-translation sumoylation	0.999
Miro1V13(+)/STRING/homology		
NEDD4-1, RPF1, KIAA0093	E3 ubiquitin protein ligase expressed in neuronal precursor cells; developmentally downregulated in the early central nervous system	0.999
Miro1N18(−)/STRING		
EF2, EEF-2, SCA26, EF-2	Member of the GTP-binding translation elongation factor family; facilitates GTP-dependent ribosomal translocation during protein synthesis	0.999
B-ALPHA-1, FLJ25113	Major microtubule component displaying enriched expression in morphologically differentiated neuronal cells	0.986
Cortactin, EMS1	Promotes polymerisation and rearrangement of the actin cytoskeleton when stimulated by external stimuli	0.985
RAB11A	Controls intracellular trafficking of the innate immune receptor TLR4. May facilitate protein trafficking. Associated with secretory pathways	0.984
Nedd-8, NEDD-8	E3 ubiquitin protein ligase expressed in neuronal precursor cells; developmentally downregulated in the early central nervous system	0.976
EPF5, E2EPF, E2-EPF	Ubiquitin conjugating enzyme E2 for targeting of proteins for proteasomal degradation	0.973
Miro1N18(−)/homology		
SMMHC, KIAA0866, AAT4, SMHC, FAA4	Myosin heavy chain 11; major contractile protein. Converts chemical energy into mechanical energy through ATP hydrolysis	N/A
Miro1N18(-)/STRING/homology		
RhoC, ARHC, RHOH9	Stimulates reorganisation of the actin cytoskeleton and regulates cell shape, attachment, and motility	0.973
PSMD4, pUB-R5, AF-1	Non-ATPase subunit of the 19S regulator lid of the 26S proteasome	0.972
Miro1N18(−)/literature		
Centromere protein F, PRO1779, CENPF	Protein that associates with the centromere-kinetochore complex; facilitates mitochondrial movement during cytokinesis	N/A

**Table 2:** Identification of 24 proteins of interest related to hMiro2 signalling

Identifier	Protein function	STRING score
Miro2V13(+)/STRING		0.999
PDHE1-B, PDHBD, PHE1B	E1 alpha 1 sub-unit of the pyruvate dehydrogenase (PDH) complex; catalyses overall conversion of pyruvate to acetyl-CoA and CO_2_. Mutations in this gene are associated with pyruvate dehydrogenase E1-alpha deficiency and X-linked Leigh syndrome	0.999
MC5DN4, COXPD22, ATP5A, ATPM, MOM2, OMR, hATP1, ATP5AL2, ORM, HEL-S-123m	ATP synthase, H + transporting, mitochondrial F1 complex, alpha subunit 1; catalyses ATP synthesis during oxidative phosphorylation	0.999
PSC5, HC5, PMSB1	Proteasome subunit beta 1. Component of the proteasome core; tightly linked to TBP (TATA-binding protein)	0.999
HDH-VIII, G3BP	G3BP stress granule assembly factor 1; facilitates DNA unwinding and acts as a signalling element of the Ras pathway, binding specifically to RasGAP	0.999
S12, P40, MOV34, MOV34L, Rpn8	A non-ATPase subunit of the 19S proteasome regulator lid of the 26S proteasome complex	0.999
EPS15R, eps15R	Epidermal growth factor receptor pathway substrate; constitutive component of clathrin-coated pits that is required for receptor-mediated endocytosis. Involved in cadherin binding associated with cell–cell adhesion	0.999
Miro2N18(−)/STRING		
S16	Ribosomal protein S16, a protein component of the 40S sub-unit	0.999
ERO1LA, Ero1alpha, ERO1-L, ERO1-L-alpha, ERO1L, ERO1-alpha	Endoplasmic reticulum oxidoreductase 1 alpha; an oxidising enzyme that exists in the endoplasmic reticulum and is induced under hypoxia	0.962
EIEE31, DNM	Member of the dynamin subfamily of GTP-binding proteins, involved in tubulation and severing of membranes and associated with clathrin-mediated endocytosis and other vesicular trafficking processes	0.942
HUWE1, KIAA1578, KIAA0312, MULE, LASU1, UREB1, URE-B1, Ib772, ARF-BP1, HECTH9, HSPC272	E3 ubiquitin ligase known to ubiquitinate the anti-apoptotic protein Mcl1 (myeloid cell leukemia sequence 1 (BCL2-related)). This protein also ubiquitinates the p53 tumour suppressor, core histones and DNA polymerase beta	0.9
VPS23, TSG10	Apparently inactive homolog of ubiquitin-conjugating enzymes. May act as a negative growth regulator. Appears important for genomic stability and cell cycle regulation	0.867
PTPN1, PTP1B	A founding member of the protein tyrosine phosphatase (PTP) family. Catalyses the hydrolysis of the phosphate monoesters specifically on tyrosine residues	0.818
hUba3, hUBA3, NAE2, UBE1C	Member of the E1 ubiquitin-activating enzyme family; associates with AppBp1, an amyloid beta precursor protein binding protein, to form a heterodimer, subsequently activating NEDD8, a ubiquitin-like protein, which regulates cell division, signalling and embryogenesis	0.81
RAP1A, RAP1, SMGP21, C21KG, KREV-1, G-22K, KREV1	Counteracts the mitogenic function of RAS through its ability to interact with RAS GAPs and RAF in a competitive manner	0.726
KIAA1794, FLJ10719	Fanconi anaemia complementation group I, associated with Fanconi anaemia	0.651
Active BCR-related, MDB	Contains a GTPase-activating protein domain; appears to play a role in vestibular morphogenesis	0.472
UBCH7, E2-F1, L-UBC, UbcM4, UBCE7	Member of the E2 ubiquitin-conjugating enzyme family known to facilitate the ubiquitination of p53, c-Fos and the NF-kB precursor p105 *in vitro*	0.471
HEL-S-70p, PURH, AICAR, IMPCHASE, AICARFT	Acts as a 5-aminoimidazole-4-carboxamide ribonucleotide formyltransferase/IMP cyclohydrolase; enhances the rate of nucleotide re-synthesis, increasing adenosine generation from adenosine monophosphate during conditions of myocardial ischemia	0.466
L23A, MDA20	Ribosomal protein L23a; may be one of the target proteins involved in mediating growth inhibition by interferon	0.999
L10A, Csa-19, CSA19, NEDD6	Ribosomal protein downregulated in neural precursor cells during development	0.999
Calnexin, CNX, IP90, P90	Calcium-binding, endoplasmic reticulum (ER)-associated protein; interacts transiently with newly synthesized N-linked glycoproteins, facilitating protein folding and assembly. Possible roles in facilitating protein folding and quality control	0.999
PTER, HPHRP, RPR-1	Phosphotriesterase-related protein; may be involved in regulation of mitosis and transport of proteins to the cell surface	0.774
CIP75, A1U, C1orf6, A1Up, UBIN	Ubiquilin 4; involved in regulation of protein degradation *via* the ubiquitin–proteasome system (UPS)	0.774
Miro2N18(−)/homology		
CASP3, CPP32B, SCA-1, CPP32, Yama, apopain	Associated with apoptotic signalling. Cleaves and activates caspases 6, 7 and 9. Is the principal caspase involved in the cleavage of amyloid-beta 4A precursor protein (a protein associated with neuronal death in Alzheimer’s disease)	N/A

**Fig. 4:**
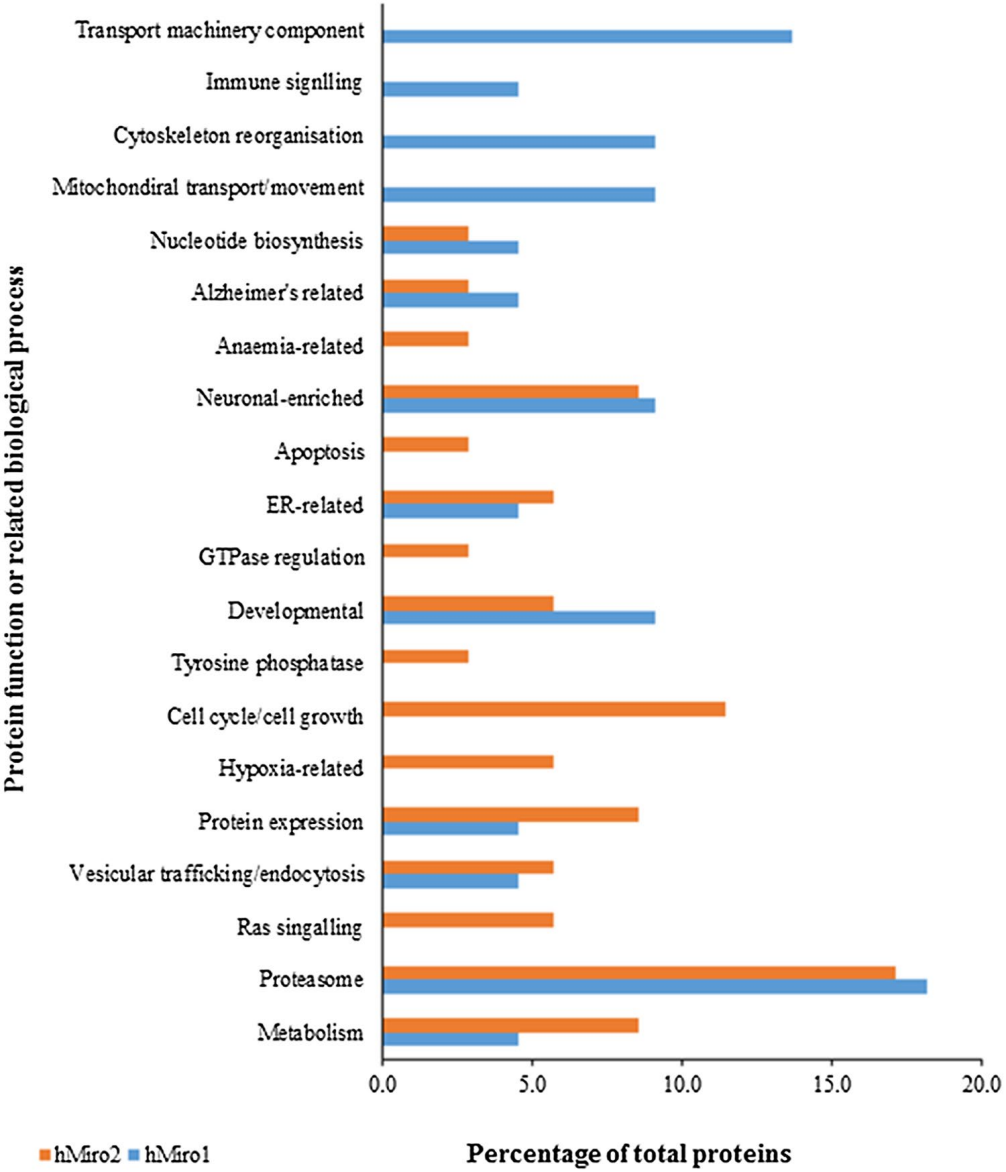
Biological processes associated with human Miro1 and Miro2. Overall biological processes that are possibly mediated by differential expression of Miro1 and Miro2 are shown using a histogram showing some distinct functions mediated exclusively by each human Miro GTPase

### hMiro1 and hMiro2 may be involved in distinct, but related, signalling pathways

The proteomic changes based on hMiro constructs transfection were analysed. The results show hMiros participating in a number of cellular function mediated by human Miros outside the mitochondrial functions. These are the overlapping functions of both hMiros where the enzymes in its GTP-bound active state can bind to a number of effectors or mediate a number of cellular processes through influencing the proteome of the cell thereby participating in a number of signalling events. The results show hMiros participating in a number of cellular function outside mitochondrial transport. When the biological functions of the proteins interacting with hMiros were analysed it was evident that proteins involved in neuronal function, proteasome-mediated functions including degradation, vesicle transport/ endocytosis, various metabolic and developmental processes were interacting with both hMiro1 and hMiro2. These are the overlapping functions of both hMiros where the GTPase in its GTP-bound active state can bind to a number of effectors involved in these processes. The differentially expressed proteins that were observed in both hMiro1 and hMiro2 active mutants show several proteasomal function genes. For hMiro1 this included many proteosomal proteins including threonyl-TRNA synthetase like 2 (TARSL-2), ribosomal protein lateral stalk subunit P0 pseudogene 6 (RPLP0P6), COP9 signalosome subunit 4 (COSP4), RNA-binding motif protein (RBM3), proteosome subunit alpha 2 (PSMA2) and neuronal precursor cell expressed developmentally downregulated 4, E3 ubiquitin protein ligase (NEDD4) (see section below), while Miro2 constitutively active mutants also show differences in the expressions of proteosome subunit beta1 (PSMB1), paraspeckle component 1 (PSPC1), ubiquitin-like modifier activating enzyme2 (UBA2), ribosomal protein S27 like (RPS27L), proteosome subunit alpha2 (PSMA2) amongst others. It is therefore evident that both hMiros cause differential expression of the proteosome synthesis, ubiquitin-mediated degradation and other proteosomal remodelling component entities when overexpressed in cells. It is important to understand that although overlapping pathways are identified in hMiros function, the cellular mediators are different depending on the specific hMiro in action.

**Fig. 5:**
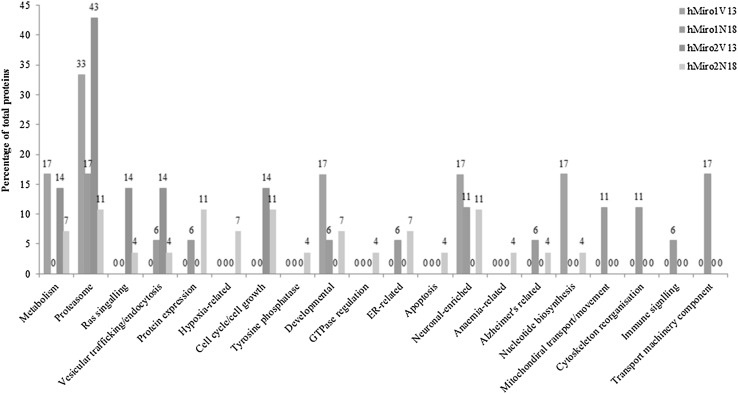
Summary of major biological functions associated with each protein of interest. Biological processes association analysed for constitutively active Miro1 (hMiro1V13) and dominant negative (hMiro1N18) are given as a percentage of total protein in grey and orange bars. Similarly, the specific biological processes associated with constitutively active Miro2 (hMiro2 V13) and dominant negative (hMiro2 N18) are given as a percentage of total protein using blue and yellow bars. (Color figure online)

When we analysed whether the two different human Miros interacted with exclusive effectors or protein-binding partners, we did identify some distinct cellular function and pathways for hMiro1 and hMiro2. The hMiro1 mediates cytoskeletal remodelling (see Figs. 4, 5) possibly through tubulin beta 2A (TBB2A_human), actin alpha 1 (ACTN1), actin-related protein 3 homolog (ACTR3), capping actin protein of muscle Z-line alpha subunit1 (CAPZA1), tropomodulin (TMOD3) and mediator of cell motility (MEMO1). However, existing literature studies already suggest the role of hMiros in mitochondrial transport through the actin cytoskeletal pathway. It is important to note that the proteins involved in transport machinery component, cytoskeletal reorganisation and mitochondrial movement may not be mutually exclusive and it is very likely that they are interconnected by function based on the cellular status. As previously stated, very little is known regarding hMiro2 cellular function in general. Therefore, we do not currently have any idea of the signalling cascade, interaction partner and GTPase effectors of hMiro2. Various novel processes that are potentially mediated by hMiro2 came to light when we analysed the proteome profile of constitutively active hMiro2. This revealed that hMiro2 could be exclusively mediated by cell cycle/cell growth function, which would relate to cancer and apoptotic functions (see Figs. 4, 5). The processes of cell death, apoptosis, cell growth and cell cycle regulation could be mediated by hMiro2 through proteins including G3BP stress granule assembly factor 1(G3BP1), heterogeneous nuclear ribonucleoprotein U (HNRNPU) nutrix hydrolase 21 protein CPSF5, nitralase family member 2 (NIT2), multifunctional protein tetratricopeptide repeat domain 38 (TTC38) and acyl-coA-binding domain containing 3 (ACBD3). It is of note that several genes involved in gene expression control such as non-POU domain containing octomer binding (NONO), histone deacetylase (HDAC1), minichromosome maintenance complex component 2 (MCM2), H3 histone family member (H3F3C) are also differentially expressed due to hMiro2 constitutive overexpression. These genes may regulate the downstream mRNA expression of other genes that control previously unspecified cellular functions.

**Fig. 6:**
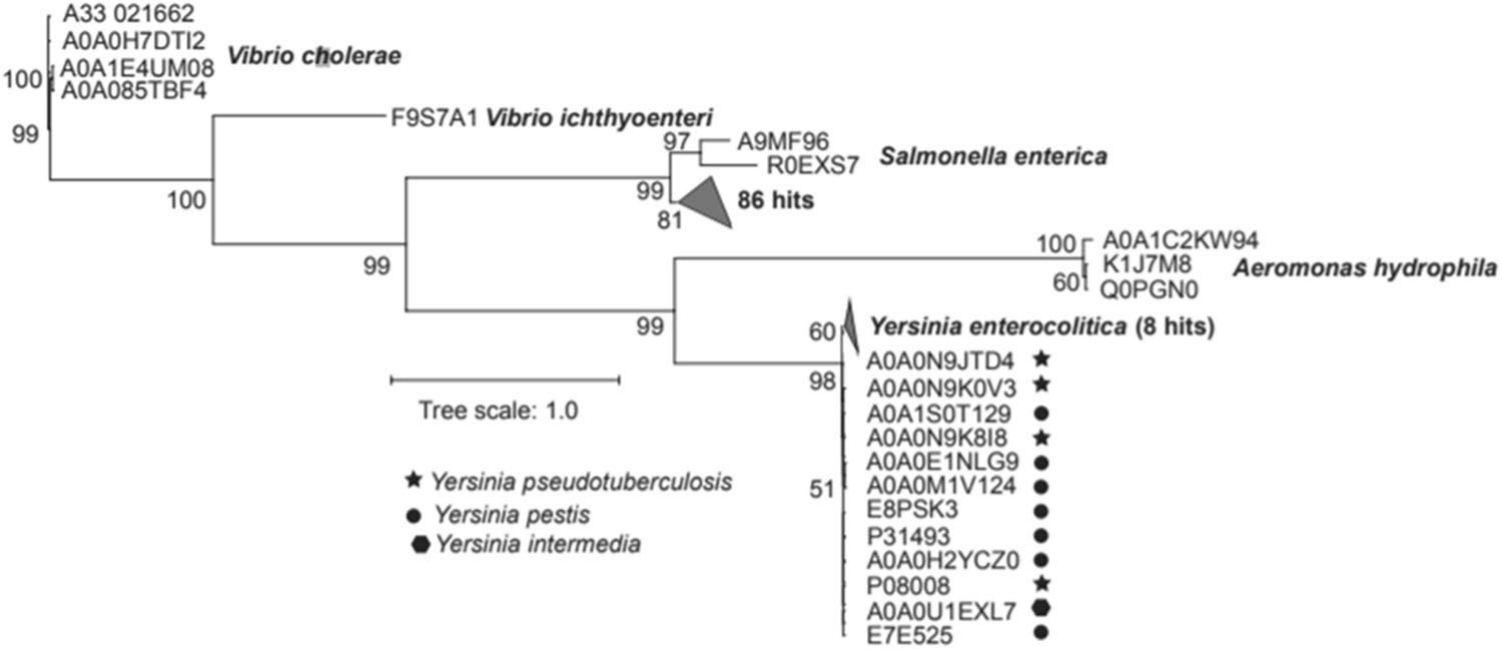
A maximum-likelihood tree derived from amino sequenced alignment of the BLAST hits (*E*-value ≤ 0.001 and > 20% sequence identity) of *Vibrio* cholerae VopE protein in the Uniprot database. The scale bar represents amino acid substitutions per site

Of the proteins of interest identified, three were particularly noteworthy: RhoC, rUB-R5 and NEDD4. These proteins were identified as potential interactors across three separate search mechanisms, including experimental analysis, homology studies and the extensive database searches performed by STRING. NEDD4 was linked to hMiro1-V13 overexpression, while RhoC and PSMD4 were associated with hMiro1-N18 over-expression. All 3 of these proteins possessed functions that showed an enriched presence in the other proteins of interest, namely the reorganisation of the cytoskeleton (RhoC, NEDD4), neuronal cell involvement (NEDD4) and links to ubiquitin-mediated proteasomal degradation (NEDD4, PSMD4). However, several groups of proteins identified were also of particular interest, including the clusters related to neuropathological disorders.

### Neuronal precursor developmentally downregulated (NEDD) proteins

The NEDD proteins were originally identified as ‘neural precursor cell expressed, developmentally downregulated’ proteins. Other than NEDD4, several other identified proteins were NEDD proteins (NEDD6, NEDD8) or proteins found to act within NEDD-related pathways (hUba3, Ras, AppB1). This implies a role for hMiros in neuronal signalling associated with proteins that are commonly downregulated in neuronal precursor cells. Intriguingly, NEDD4 was the only neurone-related protein to be associated with a V13 mutant; all of the other neuronally enriched proteins identified were associated with N18 hMiro mutants, suggesting that the status of the N-terminal GTPase domain may be important in mediating neuronally enriched signalling pathways. Indeed, while the NEDD proteins were originally identified as ‘neural precursor cell expressed, developmentally downregulated’ proteins, these proteins are frequently functionally diverse and do not necessarily occur within the same signalling pathways. Thus, while a GTP-bound version of the N-terminal GTPase domain may influence one pathway, a change of status to the GDP-bound version may influence another.

NEDD4 itself is classified as an E3 ubiquitin ligase localised to the cytoplasm, and has been demonstrated to mediate a range of important cellular processes including neuronal development, cellular growth and intracellular homeostasis [17]. NEDD4 functions through ubiquitination ligase activity and other mechanisms which are not fully understood. However, it has been established that NEDD4 can be activated by a variety of signalling molecules, and that auto-inhibition can occur in the absence of calcium [18]. Intriguingly, all of the NEDD-related proteins are developmentally downregulated in neuronal cells and many appear to play roles in normal development of the nervous system [19]. Similar functions have been noted for mammalian and fly Miro, with homozygous Miro1^−/−^ ablation in mice resulting in dysfunctional neuronal developmental, most critically resulting in incomplete lung innervation [20]. It is possible that the mammalian Miros may act within pathways that regulate NEDD4 activity, and that disruption of Miros during development prevents adequate downregulation of NEDD4 and other NEDD proteins. Conversely, a functional link between these proteins during development may not exist, and the hMiros could represent a ubiquitination target for NEDD4. Further experimental work is required to explore these possibilities. Curiously, NEDD4 has also been implicated in schizophrenia [21], a serious psychiatric condition commonly presenting with delusions, hallucinations and profound disturbances in cognition and emotion [22]. This is in congruence with the hMiro1, which has been previously linked to schizophrenia through interaction with the DISC1 multifunctional scaffolding protein [23]. Even more interestingly, DISC1 has been linked to stress-induced psychosis in schizophrenic individuals [24], a clinical feature that is presented more frequent in patients harbouring specific NEDD4 genotypes [21]. It is therefore possible that these proteins act within the same, or related, signalling pathways.

NEDD4 may also fit into the presently elusive link between Miro proteins and cellular metabolism. Prior implications in the Miro GTPases with cellular metabolism have been limited to studies in yeast [5], so the presence of several proteins related to cellular metabolism is rather intriguing, suggesting that these roles for cellular metabolism may be conserved in higher organisms. Several of the proteins of interest identified were involved in cellular metabolism, and oxidative stress has been shown to activate NEDD4 in a pathway leading to the activation of transcription factors [25]. One possibility is that signalling for excessive production of free radicals arising from oxidative metabolism occurs through Miro proteins to proteins such as NEDD4. However, this remains to be explored.

NEDD6 is perhaps less exciting, a ribosomal protein displaying developmental downregulation in neuronal precursor cells [26]. It presently appears to be involved in protein expression alone. NEDD8, however, represents an apparently multifunctional ubiquitin-like protein (ULP) sharing ∼ 60% amino acid identity with ubiquitin [27] that appears to possess tumour suppressive roles [28] and has been reported in the literature as a regulator of caspase-1 [29]. Additionally, NEDD8 has been shown to play crucial roles in endometrial function [30].

### RhoC

The suggested link between RhoC and hMiro1-N18 is also interesting, fitting into the theme that dominant negative versions of the hMiro N-terminal GTPase domain are involved in a wider range of pathways than the constitutively active versions. Rho proteins are typically associated with cytoskeletal reorganisation, although the fine-tuning of RhoC activity appears to particularly influence cell motility [31]. A link between RhoC signalling and the Miro GTPases therefore seems logical, in light of the cell migration results previously reported in studies implicating the Miros in cell motility and migration. Due to the primary localisation of RhoC at the plasma membrane [32], it is unlikely that the Miros directly interact with RhoC. However, these results indicate that hMiro1 acts downstream of the RhoC pathway in a manner that may be related to the GTPase state of the N-terminal GTPase domain.

### PSMD4 and proteasomal degradation signalling components

The notable presence of PSMD4 was initially somewhat puzzling, resisting the trend of hMiro V13 mutants being more likely to be associated with components of the proteasome complex. PSDM4 is an essential constituent of the 19S proteasomal regulatory complex [19, 33] responsible for proteasome substrate recognition and known to play important roles in sperm-zona pellucida penetration during fertilisation [34]. However, while this protein is related to the Ubiquitin proteosomal system, it is predominantly involved in ensuring that only correctly labelled (ubiquitinated) proteins are processed for degradation [19]. No components of the UBS directly linked to actual degradation were associated with hMiro N18 mutants in this study, although several proteins related to ubiquitination or the sorting of ubiquitinated proteins were. Conversely, V13 mutants were associated with both ubiquitination proteins and, in the case of hMiro2-V13, components of the proteasome protein core directly involved in proteasomal degradation. Potentially, this could indicate that Miro proteins that are active in the N-terminal GTPase domain are more likely to be ubiquitinated and subsequently degraded by the UBS, while that Miro proteins that are inactive in this domain may still be ubiquitinated but are more likely to be thoroughly checked prior to degradation. This is conjecture, however. The targeting of proteins for proteasomal degradation is complex and influenced by several signalling pathways. Thus, further investigation of the relationship between the Miro N-terminal GTPase state and propensity towards UBS-mediated degradation is required.

### Proteins involved in endoplasmic reticulum signalling

Beyond RhoC, rUB-R5 and NEDD, the two hMiro2-N18-associated proteins linked to the endoplasmic reticulum (ERO1LA and calnexin), were particularly interesting. ERO1LA, also known as endoplasmic reticulum oxidoreductase 1, pairs oxidation of thiols to the reduction of molecular oxygen to produce hydrogen peroxide (H_2_O_2_) and has been implicated in cancer progression and immune signalling [35–37]. ERO1LA has also been implicated in the regulation of Ca^2+^ fluxes at the interface between the endoplasmic reticulum and mitochondria [38]. Conversely, calnexin is a calcium-binding protein localised to contact points between mitochondria and the endoplasmic reticulum, displaying roles either as a quality control chaperon (when non-palmitoylated) or a calcium signalling protein (when palmitoylated) [39]. Interestingly, Miro proteins have been implicated in intracellular calcium buffering and have been shown to localise to ER-mitochondrial contact points. Additionally, the yeast Miro Gem1p interacts with the ER-mitochondria encounter structure (ERMES). While a direct association between Miro and ERMES in humans has not yet been established, the presence of Calnexin and another ER-related protein, ERO1A, in this study is noteworthy, suggesting that the hMiros indeed interact with ERMES-like proteins at ER-mitochondria contact points [40, 41]. Other notable groups of proteins identified in this study included proteins related to protein expression, which were enriched across various data sources and may indicate that regulation of the hMiro signalling pathways occurs, at least partly, at the level of expression. Whether the Miro proteins themselves are significantly regulated at the level of expression is unknown, though this phenomenon has been demonstrated for other atypical GTPases, such as the Rnd proteins and RhoH [42].

### Proteins associated with neurodegeneration

Finally, the presence of several Alzheimer’s and cancer-related proteins identified in this study are noteworthy. The Miro GTPases have been implicated in both neurodegenerative diseases and neoplastic disorders when dysregulated. According to the results obtained here, the development of these disorders may occur in some situations through the GTPase status of the hMiro N-terminal GTPase domain. It is uncertain why an enrichment in Alzheimer’s-related proteins was demonstrated, but not other proteins associated with neurological disorders. While Miro inhibition has been linked to Alzheimer’s through activation of the PAR-1/MARK family kinases and subsequent promotion of pathological tau phosphorylation [43], links to other neurological disorders such as Parkinson’s and ALS are repeatedly noted in the literature. The enhanced presence of Alzheimer’s-related proteins here, however, may partly reflect that Alzheimer’s is the most common form of dementia [44] and correspondingly heavily researched. Thus, proteins implicated to Alzheimer’s are more likely to be labelled as such in the literature. Regardless, the presence of proteins implicated in neoplasia and Alzheimer’s disease is in congruence with previous reports of Miro function and the development of these pathologies. Too, it is quite possible that some proteins considered ‘Alzheimer’s-related’ actually engage in other neuropathological states when dysregulated [45].

### Bioinformatics analysis for identifying VopE-like effectors in other bacteria

Although role of mitochondria in ATP generation and programmed cell death has been long established, role of mitochondria in various innate immune responses through PIG-I-like signalling, antibacterial immunity and inflammation is clearly still emerging. A study using *Vibrio cholerae* AM-19226 as a model organism as recently shown a Type 3 Secretion System (T3SS)-delivered effector VopE is required to prevent mitochondrial perinuclear clustering and to suppress innate immune responses during *V. cholerae* infection of cultured mammalian cells. This study established that Miro1 and Miro2 GTPases enzymatic activities are modulated through VopE effector binding. This interaction was shown to effectively interfere with the mitochondrial activity regulated by the Miros [16]. In a different investigation, Miro-1 was shown to control lymphocyte migration and polarity through controlling the mitochondrial accumulation around microtubule-organising centre in response to chemokines. This regulatory role of Miros over lymphocytes is highly significant since the lymphocyte migration maintaining polarity and adhesion is essential for functional immune response [46]. Therefore, it is becoming apparent that Miros play a considerable role in contributing towards the immunological role of mitochondrial. Therefore as a first step, we tried to perform bioinformatics analysis to identify possible other type III or type III-like effectors that may be modulating host immunity via Miro GTPases. The BLAST search revealed the presence of gene encoding VopE or VopE-like proteins in other strains of *Vibrio cholerae* as well as other Gram-negative bacterial species including *Aeromonas hydrophila, Salmonella enterica, Vibrio ichthyoenteri, Yersinia enterocolitica, Yersinia intermedia, Yersinia pestis* and *Yersinia pseudotuberculosis* (supplementary Fig. 1). However, the amino acid sequence similarities varied between 23.4 and 40.5% to proteins in other species.

The majority of VopE hits (88 hits; 76.5%) were from different serovars of *Salmonella enterica* that formed a distinct cluster in the ML tree (Fig. 6). Similarly, VopE sequences from all *Yersinia* species and *Aeromonas hydrophila* grouped together in distinct clades. These results suggest that the VopE sequences are quite conserved at the species level and variety of other serovars can harbour type III effectors that could modulate the Miro GTPase activity functioning as effectors or guanine nucleotide activating proteins.

We identified the conserved and semi-conserved regions in the alignment of representative sequences that were previously described by [47]. Bulge I and II regions are located from positions 203–215 and 246–260 in our alignment, respectively (Supplementary file 1). However, some of the conserved and semi-conserved sites have gaps in the alignment as the dataset analysed in this study is more diverse. The MTR domain required for mitochondrial targeting is quite diverse that includes first 92 sites in the alignment.

## Materials and methods

### Sample preparation

Mammalian pRK5-Myc primers harbouring point mutations in the hMiro1 and hMiro2 N-terminal GTPase domain were kindly donated by the Pontus Aspenström Group (Karolinska Institutet, Sweden) (Addgene plasmid No 47888). These were used to overexpress hMiro1 and hMiro2 mutants rendering the N-terminal GTPase domain either constitutively active (V13 mutants) or dominant negative (N18 mutants). Cells to be transfected for overexpression were plated onto 6-well plates at 250,000 cells per well and allowed to incubate at 37^o^C/5% CO_2_ until approximately 70% confluent. Lipofectamine™ 2000 reagent was used for the transfection of exogenous plasmid DNA (pDNA) into mammalian cells according to the manufacturer’s instructions. Cells were allowed to incubate for 24 h prior to transfection, then washed twice with PBS, allowed to trypsinize for 5 min at 37^o^C/5% CO_2_ in 0.25% trypsin–EDTA and harvested by centrifugation at 1200×*g* for 5 min. The cell pellet was subjected to lysis on ice in RIPA buffer (25 mM TRIS pH 7.4, 150 mM NaCl, 0.1% SDS, 0.5% sodium deoxycholate, 1% Triton-X) supplemented with P8340 protease inhibitor cocktail (Sigma Aldrich, UK). Each pellet was subsequently spun at 15,000×*g* for 18 min (4 °C) and the resulting supernatant retained on ice. The protein concentration of each sample was determined using Bradford’s reagent (Sigma Aldrich, UK), and 20 µg of each sample loaded twice into separate 12% SDS–PAGE gel wells, with one part of the sample assessed for overexpression success using anti-hMiro1 or anti-hMiro2 immunoblotting and the other part processed using the geLC-MS method described below.

### Proteolytic digestion

In-gel digestion was performed as previously described. Briefly, 20 µg of each sample was fractionated based on molecular weight on a 12% SDS–PAGE gel. The fractionated protein lane was cut into 8 bands and each band separately sliced into further small sections and placed into individual tubes for destaining achieved *via* 3 washes with 100 μL of 100 mM ammonium bicarbonate/acetonitrile (1:1, vol/vol) with shaking. This solution was removed, and reduction performed in 200 µL 10 mM DTT (made in 50 mM ammonium bicarbonate) at 56 °C for 60 min in a closed water bath. The gel fragments were allowed to cool, reduction solution removed and alkylation performed in the dark for 30 min using 200 µL 54 mM iodoacetamide prepared in 50 mM ammonium bicarbonate. 50 µL 100% acetonitrile was added to the gel fragments and allowed to incubate at room temperature until the gel pieces became white and shrunken. The acetonitrile was removed and the samples were subjected to drying in a centrifugal evaporator (speedvac) for 15 min to encourage dissipation of latent acetonitrile. The gel fragments were then pre-incubated on ice for 60 min with 5 µg Typsin Gold (Promega) solution prepared in 50 mM acetic acid. 20 µL ammonium bicarbonate was then added to each tube to cover gel pieces and maintain moisture during proteolytic digestion with trypsin. Trypsin digestion was performed overnight at 37 °C in a water bath with occasional gentle shaking. After at least 12 h, the digestion was arrested by addition of 100 µL 1:2 (vol/vol) 5% formic acid/acetonitrile to each tube. The tubes were then incubated with shaking for 30 min at room temperature and the supernatant retained due to the presence of extracted peptides. The addition of 30 µL of 0.1% (vol/vol) trifluoroacetic acid to each sample followed by 5-min incubation ensured further peptide extraction, the supernatant retained once again. The resulting pooled liquid was subjected to freeze-drying overnight in a Christ Alpha 1–2 LD Freeze Dryer, and the resulting lyophilised samples each re-suspended in 30 µL of LC-MS Buffer A (5% acetonitrile and 0.1% formic acid in LCMS-grade water). 6 µL of sample was loaded into a Chromacol vial and 3 µL injected for LC-MS.

### LC-MS/MS analysis on a Q-Exactive™ hybrid quadrupole-orbitrap mass spectrometer

The 8 fractions for each sample were subjected to LC-MS/MS analysis on a Q-ExactiveTM Hybrid Quadrupole-Orbitrap Mass Spectrometer (Thermo Scientific, Bremen, Germany) connected to an Ultimate 3000 nano LC-MS/MS system (Dionex LC packings, Amsterdam, The Netherlands). Separation of peptides was accomplished with an EasySpray column (PepMap C18, 2 µm 100 A, 75 µm x 50 cm) maintained at 45 °C. The peptide fractions were loaded onto the trap column at a flow rate of 3 ml min^− 1^ using 0.1% formic acid/5% acetonitrile (solvent A). The peptides were then fractionated on an analytical column using an LC gradient of 5–40% of solvent B (95% ACN, 0.1% formic acid) over a period of 120 min at a flow rate of 0.2 µL min^− 1^. The spray voltage was set to 2.3 kV. Data were obtained using a data-dependent Top 10 methodology, with scans acquired in the Orbitrap mass analyser over a range of 150–1500 m/z, mass resolution set to 70,000 and target value set to 1.00E + 06. The ten most intense peaks with a charge state of ≥ 2 were subsequently fragmented in the HCD collision cell and tandem mass spectra obtained in the Orbitrap with mass resolution set to 17,500 and target value set to 1.00E + 05. Maximum ion accumulation times were 100 ms for the full MS scan and 50 ms for the tandem mass spectra. Polydimethylcyclosiloxane (m/z, 445.1200025) ions were used for internal calibration.

### Data analysis

Mass spectrometry data were searched against the NCBI Human RefSeq 70 database, using X!Tandem and MASCOT search platforms. Carbamidomethylation of cysteine was set as a static modification, while the oxidation of methionine was set as a dynamic modification. Other search parameters included the allowance of one missed cleavage by trypsin and a 1% false discovery rate (FDR) at PSM level. Terms for biological processes and molecular functions were derived from NCBI RefSeq and the Human Protein Reference Database (HPRD). Progenesis QI, compatible with MASCOT search results, was used to align ion intensity maps and resolve conflicts. The data obtained from proteomic analysis before superimposition on bioinformatics tools were also analysed using progenesis software-based IMPaLA (Integrated Molecular Pathway Level Analysis) online tool. For further analysis, data were compared to predicted interaction partners for hMiro1 and hMiro2 according to the STRING 2.0 protein–protein interaction prediction database. Comparisons were also drawn to known interactors according to manual literature searches, and proteins known or predicted to interact with the closest homologues of hMiro1 and hMiro2 according to the STRING 2.0 database.

### VopE sequence analyses using bioinformatics tools

The amino acid sequence of VopE (locus tag: A33_021662) was extracted from the genome of *Vibrio cholerae* strain AM-19226 (Accession number: AATY02000000). The sequence was BLAST searched [48] in the UniProt database. The protein sequence of 115 hits with *E*-value ≤ 0.001 and > 20% sequence identity (Supplementary Table 1) were aligned to the query sequence using CLUSTLW [49].

A maximum-likelihood (ML) tree was generated from this alignment following the JTT + G4 substitution model with 100,000 SH-aLRT (SH-like approximate likelihood ratio tests) and 100,000 ultrafast bootstrap iterations using IQ-Tree [50, 51]. The tree was visualised using iTOL [52].

## Conclusions

The hMiro GTPases are important enzymes that play significant roles in mitochondrial transport and homeostasis, and may be significantly involved in the development of several neuropathological disorders. Proteome characterisation of the effects of hMiro1 and hMiro2 GTPase states on intracellular signalling through functional assays and validation will aid in the understanding of the roles of these important mitochondrial enzymes in health and disease conditions. However, our initial proteomic and bioinformatics analyses clearly indicate that there are mutually exclusive cellular processes that are mediated by individual Miros. While Miro1 is seen to be mediating cellular function through modulation of signalling pathways that mediate Transport machinery components, immunological responses, cytoskeletal reorganisation and mitochondrial movement, the Miro2-specific signalling mediation can be classified into apoptosis, Hypoxia, Ras pathway regulation and tyrosine phosphatase-mediated signalling cascades. A number of proteins involved in redox pathways and uncharacterised proteins with putative function are identified in this study for which any specific function and signalling pathway cannot be attributed with confidence at this stage. Scaffold proteins such as DLG1 or 14-3-3 proteins that are identified to be significantly upregulated may indicate that hMiros participate in larger protein assemblies. Based on the domain architecture and previously reported functions of hMiro and its homologues, it is tempting to propose that hMiros may form a number of protein assemblies mediateing varying subcellular functions based on cellular statuses such as ATP requirement, calcium concentration or redox state of the cell. Although we have identified specific protein entities that could function as the effectors of these GTPase or simply interactors of large protein assembly in complex cellular processes it will be essential to experimentally validate the co-expression, binding or signal cascading using experimental approaches in future. We predict that the identification of numerous putative hMiro1 and hMiro2 interactors and signalling pathways here will aid in developing a more complete picture of the range of molecular functions and processes associated with these enzymes, paving the way for future proteomic studies to help further elucidate Miro-related signalling pathways and their influence on health and pathology.

## Electronic supplementary material

Below is the link to the electronic supplementary material.

Supplementary material 1 (PDF 8 KB)

Supplementary material 2 (PNG 130 KB)

## Abbreviations

ACN: Acetonitrile
FDR: False discovery rate
GO: Gene ontology
hMiro: Human Miro GTPase
PBS: Phosphate buffered saline
PSM: Peptide-to-spectrum match
