# Supplementary material for: Proteomics and bioinformatics analyses identify novel cellular roles outside mitochondrial function for human miro GTPases
Source: Mol Cell Biochem. 2018 Jun 25;451(1):21–35. doi: 10.1007/s11010-018-3389-6 (PMC6342832; doi:10.1007/s11010-018-3389-6)
Supplement: Supplementary file 1 — Supplementary material 1 (PDF 8 KB) [file 11010_2018_3389_MOESM1_ESM.pdf]

CLUSTAL W (1.8) multiple sequence alignment (BioEdit-generated mock-up)

```
A33_021662_Vibrio_cholerae_AM19226  --------------------------------------------------
F9S7A1_Vibrio_ichthyoenteri         --------------------------------------------------
A9MF96_Salmonella_arizonae          ---------------------MTSNTRLYIAKENTNEAHVAPEKFASKVL
T2Q0P4_Salmonella_enterica          MLKYEERKLNNLTLSSFSKVGVSNDARLYIAKENTDKAYVAPEKFSSKVL
R0EXS7_Salmonella_enterica          --------MNNLTLSSFSKTGLPSDTRLYIAKDNSRETYVAPEKFASKVL
A0A1C2KW94_Aeromonas_hydrophila     --------------------------------------------------
A1JUA9_Yersinia_enterocolitica      --------------------------------------------------
E7E525_Yersinia_pestis              --------------------------------------------------
P08008_Yersinia_pseudotuberculosis  --------------------------------------------------
A0A0U1EXL7_Yersinia_intermedia      --------------------------------------------------

A33_021662_Vibrio_cholerae_AM19226  -----------MKVLQRTCNYIRSN--------LNQSISRLNVAHIRNVK
F9S7A1_Vibrio_ichthyoenteri         ----------------------------------------LNHDKINNGF
A9MF96_Salmonella_arizonae          TWLGRVPLFKNIDAVQKHMENTRVQNQKTLQVFLKALTEKYDEKSVNAIT
T2Q0P4_Salmonella_enterica          TWLGKMPLFKNTEVVQKHTENIRVQDQKILQTFLHALTEKYGETAVNDAL
R0EXS7_Salmonella_enterica          TWLGNIPLFKNIAAVQKHTENTRVQDQKSLQVFLKALTEKYDEKTINAVT
A0A1C2KW94_Aeromonas_hydrophila     --MQIQTHTGGLQAVAQHSDAAAGVGKFGQLDARQVATSQDVLQLGSRSE
A1JUA9_Yersinia_enterocolitica      --MKISSFISTSLPLPTSVSGSSSVGEMSGRSVSQQKSEQYANNLAGRTE
E7E525_Yersinia_pestis              --MKISSFISTSLPLPTSVSGSSSVGEMSGRSVSQQTSDQYANNLAGRTE
P08008_Yersinia_pseudotuberculosis  --MKISSFISTSLPLPTSVSGSSSVGEMSGRSVSQQTSDQYANNLAGRTE
A0A0U1EXL7_Yersinia_intermedia      --MKISSFISTSLPLPTSVSGSSSVGEMSGRSVSQQTSDQYANNLAGRTE

A33_021662_Vibrio_cholerae_AM19226  NERPIS-----ISVNAEANFFEGVSILNKPRTRVSNMR------QLLPLE
F9S7A1_Vibrio_ichthyoenteri         SCKVFN-----VKQSNENKYHK---------ERISPQR------KQLPLE
A9MF96_Salmonella_arizonae          LMAGLNDSIKPFTPVRLQQITQMVKDAEESFSKDIRSKQNASLPKVFSLV
T2Q0P4_Salmonella_enterica          LMSRIN-MNKPLTQRLAVQITECVKAADEGFINLIKSKDNVGV-RNAALV
R0EXS7_Salmonella_enterica          VVAGLNDTIKPLTPARIQQIKKMAENAKEGFSKDIMSKQHVGLPKHFSLV
A0A1C2KW94_Aeromonas_hydrophila     PQKGEG-----LLSRLGAQLARPFVALKEWIGNLLGAR-------PAAPV
A1JUA9_Yersinia_enterocolitica      SPQGSS-----LASRITEKLSSMAHSAIEFIKRMFSEG-------SHKPV
E7E525_Yersinia_pestis              SPQGSS-----LASRIIERLSSVAHSVIGFIQRMFSEG-------SHKPV
P08008_Yersinia_pseudotuberculosis  SPQGSS-----LASRIIERLSSVAHSVIGFIQRMFSEG-------SHKPV
A0A0U1EXL7_Yersinia_intermedia      SPQGSS-----LASRIIERLSSVAHSVIGFIQRMFSEG-------SHKPV

A33_021662_Vibrio_cholerae_AM19226  SFERISKAYE-SKDVRLVA----RDSAFLGLQRAI-RSERFELDNFKSNF
F9S7A1_Vibrio_ichthyoenteri         IFSKNEKLLEKHMNHHQIG----LNSAFMGIERAL-NCELFTAESLKIDF
A9MF96_Salmonella_arizonae          AKGVETKVTEQNGDFGTGMTQLLLDIALNGVKRAIPQLEKVDGNSLRKNF
T2Q0P4_Salmonella_enterica          IKGGDTKVAEKNNDVGAESKQPLLDIALKGLKRTLPQLEQMDGNSLRENF
R0EXS7_Salmonella_enterica          VKGGEIKVPEQNVDADALKTQMLLDIAVNGLKRTIPQLEKVDGKSLRENF
A0A1C2KW94_Aeromonas_hydrophila     RS-APPADNLSLADQKRLLLQKALPFTLGGLDKASELNN~IDAQQLGQEH
A1JUA9_Yersinia_enterocolitica      VTPAPTPAQMPSPTSFSDSIKQLAAETLP---KYMQQLSSLDAETLQKNH
E7E525_Yersinia_pestis              VTPAPTPAQMPSPTSFSDSIKQLAAETLP---KYMQQLNSLDAEMLQKNH
P08008_Yersinia_pseudotuberculosis  VTPAPTPAQMPSPTSFSDSIKQLAAETLP---KYMQQLNSLDAEMLQKNH
A0A0U1EXL7_Yersinia_intermedia      VTPAPTPAQMPSPTSFSDSIKQLAAETLP---KYMQQLNSLDAEMLQKNH

                                           G LR   T                                Q G
A33_021662_Vibrio_cholerae_AM19226  PYLTVANGSLRTIVTGLKGIVEFDDG-QMKDIAKEILDTQICGVPFSQFG
F9S7A1_Vibrio_ichthyoenteri         RGLATNNGSLRAMATGLQGLVQFGDC-KTQRLAEELLNKQIQSIPFSQFG
A9MF96_Salmonella_arizonae          REMASGNGPLRTLMTNLQNLSLVPEVKQLNDYAINLKNIQVGTAPFSQWG
T2Q0P4_Salmonella_enterica          QEMASGNGPLRSLMTNLQNLNKIPEAKQLNDYVTTLTNIQVGVARFSQWG
R0EXS7_Salmonella_enterica          HAMSSGNGALRSLMTNLSNLKYIPEAKQLNDDAIKLKEIPVGVALFSQWG
A0A1C2KW94_Aeromonas_hydrophila     ARLATGNGALRSLATSLNGIKDGSMRQESQTLAAGLLERPIAGIPLQQWG
A1JUA9_Yersinia_enterocolitica      DQFATGSGPLRGSITQCQGLMQFCGG-ELQAEASAILNTPVCGIPFSQWG
E7E525_Yersinia_pestis              DQFATGSGPLRGSITQCQGLMQFCGG-ELQAEASAILNTPVCGIPFSQWG
P08008_Yersinia_pseudotuberculosis  DQFATGSGPLRGSITQCQGLMQFCGG-ELQAEASAILNTPVCGIPFSQWG
A0A0U1EXL7_Yersinia_intermedia      DQFATGSGPLRGSITQCQGLMQFCGG-ELQAEASAILNTPVCGIPFSQWG

                                    T  G
A33_021662_Vibrio_cholerae_AM19226  TCSGSARDLVDNAS-------YQQEKIIIKHLNELFEKVALHLVGAEV--
F9S7A1_Vibrio_ichthyoenteri         TYSGVAAERIATA-------------------------------------
A9MF96_Salmonella_arizonae          TCGGEVARWIDKASDQELTLAAKKIQVIVEKLKYVATELENIKAGAPMSQ
T2Q0P4_Salmonella_enterica          TCGGEVERWVDKASTHELTQAVKKIHVIAKELKNVTAELEKIEAGAPMPQ
R0EXS7_Salmonella_enterica          TNGGEVAKWIDKASDQELTLAAKSIQTILKEVQKIATELSNIKIGAPVLQ
A0A1C2KW94_Aeromonas_hydrophila     TVGGKVTELIANATPEQLQEAMSQLHAVMAEVADLQRAVKAEVAGEPLPA
A1JUA9_Yersinia_enterocolitica      TIGGAASAYVASG--VDLTQAANELKGLAQQMHQLLSLM-----------
E7E525_Yersinia_pestis              TIGGAASAYVASG--VDLTQAANEIKGLAQQMQKLLSLM-----------
P08008_Yersinia_pseudotuberculosis  TIGGAASAYVASG--VDLTQAANEIKGLAQQMQKLLSLM-----------
A0A0U1EXL7_Yersinia_intermedia      TIGGAASAYVASG--VDLTQAANEIKGLAQQMQKLLSLM-----------

A33_021662_Vibrio_cholerae_AM19226  --------------------------------------------------
F9S7A1_Vibrio_ichthyoenteri         --------------------------------------------------
A9MF96_Salmonella_arizonae          R-------------------------------------------------
T2Q0P4_Salmonella_enterica          TMSGPTLGLARFAVSSIPINQQTQVKLSDGMPVPVNTLTFDGKPVALAGS
R0EXS7_Salmonella_enterica          S-------------------------------------------------
A0A1C2KW94_Aeromonas_hydrophila     TTNAEVVVAPYGEAKPAARETVTMARQTEVTGYKQALELISYQASYLLRD
A1JUA9_Yersinia_enterocolitica      --------------------------------------------------
E7E525_Yersinia_pestis              --------------------------------------------------
P08008_Yersinia_pseudotuberculosis  --------------------------------------------------
A0A0U1EXL7_Yersinia_intermedia      --------------------------------------------------

A33_021662_Vibrio_cholerae_AM19226  --------------------------------------------------
F9S7A1_Vibrio_ichthyoenteri         --------------------------------------------------
A9MF96_Salmonella_arizonae          --------------------------------------------------
T2Q0P4_Salmonella_enterica          YPKNTPDALAAHMKMLLEKECSCLVVLTSEDQMQAKQLPPYFRGSYTFGE
R0EXS7_Salmonella_enterica          --------------------------------------------------
A0A1C2KW94_Aeromonas_hydrophila     QASTEVTLSSDDLNALHQHIADGSINGSHMAKLQTRGDLQILR---TLAL
A1JUA9_Yersinia_enterocolitica      --------------------------------------------------
E7E525_Yersinia_pestis              --------------------------------------------------
P08008_Yersinia_pseudotuberculosis  --------------------------------------------------
A0A0U1EXL7_Yersinia_intermedia      --------------------------------------------------

A33_021662_Vibrio_cholerae_AM19226  --------------------------------------------------
```

```
F9S7A1_Vibrio_ichthyoenteri         --------------------------------------------------
A9MF96_Salmonella_arizonae          --------------------------------------------------
T2Q0P4_Salmonella_enterica          VHTNSQKVSSASQGEAIDQYN----MQLSCGEKRYTIPVLHVKNWPDHQP
R0EXS7_Salmonella_enterica          --------------------------------------------------
A0A1C2KW94_Aeromonas_hydrophila     SLAGGSDANGASLGNALDSLASARPNQRLVLGGLMQFAGQTDQAWADQTA
A1JUA9_Yersinia_enterocolitica      --------------------------------------------------
E7E525_Yersinia_pestis              --------------------------------------------------
P08008_Yersinia_pseudotuberculosis  --------------------------------------------------
A0A0U1EXL7_Yersinia_intermedia      --------------------------------------------------

A33_021662_Vibrio_cholerae_AM19226  --------------------------------------------------
F9S7A1_Vibrio_ichthyoenteri         --------------------------------------------------
A9MF96_Salmonella_arizonae          --------------------------------------------------
T2Q0P4_Salmonella_enterica          LPSTDQLEYLADRVK-NSNQNGAPGRSSSDKHLPMIHCLGG--VGRTGTM
R0EXS7_Salmonella_enterica          --------------------------------------------------
A0A1C2KW94_Aeromonas_hydrophila     GKPEDRLDAGARLRFDTGHMKAELARLDDSAARQVLLQLEGDFGDRAKAV
A1JUA9_Yersinia_enterocolitica      --------------------------------------------------
E7E525_Yersinia_pestis              --------------------------------------------------
P08008_Yersinia_pseudotuberculosis  --------------------------------------------------
A0A0U1EXL7_Yersinia_intermedia      --------------------------------------------------

A33_021662_Vibrio_cholerae_AM19226  --------------------------------------------------
F9S7A1_Vibrio_ichthyoenteri         --------------------------------------------------
A9MF96_Salmonella_arizonae          --------------------------------------------------
T2Q0P4_Salmonella_enterica          AAALVLKDNPHSNLEQVRADFRNSRNNRMLEDASQFVQLKAMQAQLLMTT
R0EXS7_Salmonella_enterica          --------------------------------------------------
A0A1C2KW94_Aeromonas_hydrophila     CDFAVAQVSTFADSESSPEAVLVSRLTRMGNLVGSLTDELKVRLQLPESA
A1JUA9_Yersinia_enterocolitica      --------------------------------------------------
E7E525_Yersinia_pestis              --------------------------------------------------
P08008_Yersinia_pseudotuberculosis  --------------------------------------------------
A0A0U1EXL7_Yersinia_intermedia      --------------------------------------------------

A33_021662_Vibrio_cholerae_AM19226  --------------------------------
F9S7A1_Vibrio_ichthyoenteri         --------------------------------
A9MF96_Salmonella_arizonae          --------------------------------
T2Q0P4_Salmonella_enterica          AR------------------------------
R0EXS7_Salmonella_enterica          --------------------------------
A0A1C2KW94_Aeromonas_hydrophila     RGEPTMIDSVSQLTPLELAALAHIGVEAGYLE
A1JUA9_Yersinia_enterocolitica      --------------------------------
E7E525_Yersinia_pestis              --------------------------------
P08008_Yersinia_pseudotuberculosis  --------------------------------
A0A0U1EXL7_Yersinia_intermedia      --------------------------------
```
