# Supplementary figures and images for: Proteomics and bioinformatics analyses identify novel cellular roles outside mitochondrial function for human miro GTPases
Source: Mol Cell Biochem. 2018 Jun 25;451(1):21–35. doi: 10.1007/s11010-018-3389-6 (PMC6342832; doi:10.1007/s11010-018-3389-6)

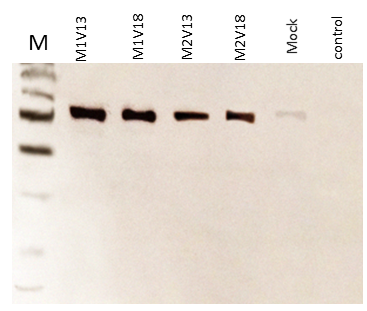

Supplement: Supplementary file 2 — Supplementary material 2 (PNG 130 KB) [file 11010_2018_3389_MOESM2_ESM.png]
